# Influence of the COVID-19 Outbreak on Disease Activity and Quality of Life in Inflammatory Bowel Disease Patients

**DOI:** 10.3389/fpsyt.2021.664088

**Published:** 2021-04-22

**Authors:** Chiara Conti, Ilenia Rosa, Luigia Zito, Laurino Grossi, Konstantinos Efthymakis, Matteo Neri, Piero Porcelli

**Affiliations:** ^1^Department of Psychological, Health, and Territorial Sciences, University “G. D'Annunzio” of Chieti-Pescara, Chieti, Italy; ^2^Department of Dynamic and Clinical Psychology, and Health Studies, “Sapienza” University of Rome, Rome, Italy; ^3^Gastroenterology Unit, Spirito Santo Hospital, Pescara, Italy; ^4^Department of Medicine and Aging Sciences, ‘G. D'Annunzio' University of Chieti-Pescara, Chieti, Italy

**Keywords:** COVID-19, disease activity, inflammatory bowel disease, health-related quality of life, outbreak, psychological distress

## Abstract

**Objective:** The present preliminary cross-sectional study aimed to investigate the extent to which health-related quality of life of patients with inflammatory bowel disease (IBD) was influenced by the outbreak of Covid-19 while controlling for disease activity.

**Methods:** Two samples of 195 (recruited before Covid-19 outbreak) and 707 patients (recruited during the Covid-19-related lockdown) were included. Psychological distress (Hospital Anxiety and Depression Scale, HADS), quality of life (Inflammatory Bowel Disease Questionnaire, IBDQ), and somatization (Patient Health Questionnaire, PHQ-12) were concurrently assessed.

**Results:** Patients with active IBD were more prevalently affected by ulcerative colitis (60.2%, η^2^ = 0.12) and, expectedly, showed higher psychological distress (HADS, d = 0.34) and somatization (PHQ-12, d = 0.39), as well as poorer disease-specific health-related quality of life (effect sizes for the total and subscale IBDQ scores in the large range of d > 0.50). Hierarchical regression models revealed that setting (pre-Covid-19 outbreak *vs*. during lockdown) (*p* < 0.001) explained only a small portion (8%) of the IBDQ variance. IBD-related factors (ulcerative colitis and disease activity) and psychological factors (psychological distress and somatization) added a significant amount of 25 and 27%, respectively, to the explained IBDQ variance. The final model predicted 59% of the explained IBDQ variance.

**Conclusion:** Clinical and psychological manifestations seem to be major impairments in IBD patients both before and during the Covid-19 outbreak. Furthermore, the quality of life of IBD patients seem to be more influenced by psychological and somatizing distressing symptoms than the pandemic-related living conditions.

## Introduction

The WHO-declared Covid-19 pandemic in March 2020 (1) is affecting severely the health, safety, and well-being of individuals, particularly of the fragile population (e.g., poor, chronically ill, elderly, health-care workers) (2). Chances of getting the virus and limited access to medications stemming from this global pandemic make the management of the chronically ill an urgent public health emergency (3).

Chronic somatic diseases are bidirectionally related to psychological distress. From one side, acute as well as chronic psychological stress may trigger disease relapses through neuroendocrine and inflammatory mechanisms (4), and, from the other side, chronic disease may represent a stable stressful condition that activates subjective distress and health concerns through allostatic overload (5). The link between the course of medical illnesses and catastrophic events has been consistently demonstrated in natural disasters as earthquakes in different regions of the world such as cardiovascular, respiratory, and gastrointestinal diseases in Japan (6, 7) and Italy (8, 9).

Inflammatory bowel disease (IBD) includes a set of chronic intestinal diseases of unknown etiology (probably a combination of genetic predisposition and an abnormal immune response) consist of ulcerative colitis (UC) and Crohn's disease (CD) as more common conditions. It is characterized by intermittent severe relapses (the predominant clinical symptoms being acute abdominal pain, blood in the stools, and acute diarrhea) and periods of quiescence. During the course of the disease, IBD patients might have acute extraintestinal symptoms, such as skin, ocular, and joint disorders, that make necessary hospitalization and intensive care and consequently this is a risk of cancer. Due to unforeseeable course, the disease may be life threatening. The incidence and prevalence of IBD has increased since the middle of 20th century worldwide and it is estimated that more than three million of individuals is affected by IBD in Europe (10). Because of the bidirectional brain-gut axis, psychological distress and disease activity showed to be consistently linked bidirectionally over time. For example, a large 2-years longitudinal study on 405 IBD patients showed a 6-fold increase of later anxiety in patients with baseline disease activity and, in turn, patients with higher baseline anxiety had a 2-fold increase of later flares-up (11).

The safety and management of patients with IBD during the pandemic are of particular concern, especially because of limited access to medications and specialistic visits, thus increasing the risk of relapses (12). Covid-19 has redefined the delivery of medical care not critical and not urgent procedures are rescheduled, and social distancing measures have led to rapid increasing of telemedicine (13).

Furthermore, stressful events and high perceived stress are well-known factors that may contribute to increase psychological distress, to impair health-related quality of life (HRQL), and even to trigger disease relapses in IBD patients (14). In the last decades, both research studies and drug authorities as the Food and Drug Administration have strongly supported the use of Patient-Reported Outcomes (PROs) measures, highlighting the relevance of relying on patients' own experience rather than exclusively on a clinician-based outcome assessment for a comprehensive evaluation of clinical health status (15, 16). Recently, a few studies have used PROs measures for investigating the effects of the Covid-19 on the clinical status of patients with physical problems (17). Due to the above-mentioned problems in medical disease management (12), patients reported a 20–30% prevalence of symptoms of anxiety and depression as well as poor HRQL (18). Other investigations reported that disability and HRQL appears to be unaffected by the Covid-19 during the stay-at-home phase of the pandemic only but without evaluating the burden of concomitant disease activity (19, 20).

The Covid-19 pandemic is a totally new catastrophic event in the world and its impact on the psychological health status of IBD patients is still under investigation. Of interest, data on the IBD population four and 24 months after the Great East Japan Earthquake in March 2011 showed different time-lag effects. Life-event stress induced by the earthquake was associated with higher relapse rates in UC but not CD patients mediated by changes in diet and family finances worries (21). In contrast, relapse rates decreased significantly at the 2-years follow-up, even though time-to-event analyses showed a significant 2-fold increase of needed additional medical treatment in patients who had experienced the death of family members or friends (22).

The present preliminary cross-sectional study aimed to investigate the extent to which HRQL of IBD patients might be influenced by the outbreak of Covid-19 while controlling for disease activity. Based on earlier findings (14, 18, 19, 23), we expected that disease activity would be associated with psychological distress and probably the stressful stay-at-home period and that the pandemic outbreak could represent an additional independent factor of psychological distress affecting HRQL.

## Methods

### Participants and Procedure

Two groups of IBD patients were included in the study. The first group was formed of 224 IBD outpatients consecutively recruited before the Covid-19 outbreak in hospital-based secondary care centers in a region of central Italy (Abruzzo) from June to December 2019. The second group included 985 Italian IBD patients recruited through an online survey (www.qualtrics.com/it) during the highest pandemic peak and stay-at-home period in Italy from March to May 2020. Patients in both groups were excluded if had been documented or self-reported comorbid severe medical, psychiatric or neurological disorders in the last 10 years or were unable to complete the self-rating scale. In the second group, patients were excluded if had been positive for SARS-CoV-2 infection/Covid-19 disease. To maximize the ecological validity of the sample, in both groups, patients from 18 to 75 years old with UC or CD were included. Furthermore, patients in the second online group were included if they received a specialistic visit by their gastroenterologist within the last 6 months and their disease activity index score was available in their medical record (see Section Sociodemographic and IBD-Related Clinical Variables below).

All patients provided written informed consent to participate in the study. The study was approved by the local ethics committee.

### Measures

#### Sociodemographic and IBD-Related Clinical Variables

Sociodemographic and IBD-related clinical variables were self-reported by the participants, including gender, educational level, and disease characteristics. Disease characteristics included the type of disease (UC or CD) and disease activity (active or quiescent) reported in the last medical visit.

In the first at-site group, disease activity was assessed by standard indices during medical visits.

In UC patients, the disease activity was measured using the full Mayo score (24). Full Mayo score is a clinical index that is composed of four clinical parameters, each scored on a 4-point Likert response scale: (1) stool frequency (0 = within individual normal frequency to 3 = at least five times more than normal), (2) rectal bleeding (0 = none to 3 = only blood evacuations), (3) endoscopic findings (0 = normal rectal mucosa to 3 = severe bleeding), and (4) physician's global assessment (0 = normal health status to 3 = severe poor global health and functional impairment). The total scale score ranges from 0 to 12 and can be categorized into four disease activity groups: 0–2 remission, 3–5 mild, 6–10 moderate, and 11–12 severe activity (25). In the current study, the Mayo score was used to classify patients with active (Mayo score > 2) vs. quiescent (Mayo score ≤ 2) UC.

In CD patients, the disease activity was measured using the Crohn's Disease Activity Index (CDAI) (26). CDAI is results from the weighted sum score of eight clinical variables (number of liquid or very soft stools, abdominal pain, general well-being, extraintestinal complications, antidiarrheal drugs, abdominal mass, hematocrit, deviation from standard body weight) The total score ranges from 0 to 600 and can be categorized into 4 disease activity groups: <150 remission, 150–219 mild, −450 moderate, and > 450 severe activity (27, 28). In the current study, the CDAI score was used to classify patients with the active (CDAI score ≥ 150) vs. quiescent (CDAI score <150) CD.

In the second online group, disease activity was reported by patients based on their last gastroenterological visit (see Section Participants and Procedure above).

#### Psychological Distress

The self-report 14-item Hospital Anxiety and Depression Scale (HADS) was used to assess psychological distress (29). The HADS is composed of two distinct seven-item subscales for depression and anxiety and is particularly suited for medical patients because it does not include overlapping somatic items. In this investigation, a total score was calculated for overall psychological distress that may vary from 0 (absence of symptoms) to 42 (high symptoms). Within this sample, Cronbach's α was 0.88 for the total scale, 0.88 for anxiety, and 0.71 for depression subscales.

#### Health-Related Quality of Life

The self-report 32-item Inflammatory Bowel Disease Questionnaire (IBDQ) was used to assess the HRQL in IBD (30). The IBDQ is composed of four areas: bowel symptoms (10 items), emotional function (12 items), systemic symptoms (five items), social function (five items), and is specifically designed for patients with IBD. The total score ranges from 32 to 224, a higher score corresponds to a better HRQL. Within this sample, Cronbach's α was 0.95.

#### Somatization

The self-report 12-item Patient Health Questionnaire (PHQ-12) (31) was used to assess somatization. The instrument is a modified version of the widely used Patient Health Questionnaire-15 (PHQ-15) (32) that excluded three gastrointestinal symptoms. The PHQ-12 includes a list of 12 symptoms (e.g., fatigue, pain) that account for more than 90% of the physical complaints reported in primary care (32). Within this sample, Cronbach's α was 0.83.

### Statistical Analysis

Student's *t*-test and chi-square test (χ^2^) were used to evaluated differences between patients with active and quiescent disease activity concerning clinical and sociodemographic characteristics. The effect size was measured using the standardized mean difference (Cohen's d) in the student's *t*-test. Cohen's d values between 0.20 and 0.50 represent small effect size, 0.50–0.80 moderate, and >0.80 large (33). The Eta-squared (η^2^) was used for the measure of effect size in the chi-square test. A standardized effect size of 0.01–0.05 is considered small, 0.06–0.14 moderate, and >0.14 large.

Hierarchical regression analysis was executed to determine major factors that best predict the HRQL. The HRQL was the dependent variable, whereas the independent variables were setting, age, gender, disease, disease activity, and psychological symptoms. Three regression models were estimated, and regression coefficients and the corresponding *p*-values were calculated. In the first model, the setting was entered to evaluate the contribution of the Covid-19 pandemic. Age and gender were entered in the second model. To estimate separately IBD-related clinical and psychological variables, disease and disease activity were included in the second model too, and distress and somatization were added in the third model.

All statistical analyses were computed using IBM SPSS Statistics version 25 (34).

## Results

Before Covid-19 (first group), 224 patients were screened, 195 (87.1%) of whom were included, while during Covid-19 (second group), 985 patients were screened and 707 (71.8%) were included. In total 902 patients were enrolled (71.2% women, mean age 40.07 ± 12.97, mean education 13.63 ± 3.56 years, 53.9% with UC). Active disease was recorded or self-reported by 407 (45.1%) patients. Compared to quiescent patients, those with active disease were affected prevalently by UC (60.2%) than CD (39.2%) (η^2^ = 0.12) and, expectedly, showed higher HADS score for psychological distress (d = 0.34) and PHQ-12 score for somatization (d = 0.39), as well as poorer disease-specific HRQL (IBDQ, effect sizes for the total and subscale scores were in the large range of d > 0.50) (Table 1).

**Table 1:** Sociodemographic and clinical characteristics of patients.

	**Disease activity**				
	**Active *N* = 407**	**Quiescent *N* = 495**	**t/ χ^2^**	**p**	**d/ η^2^**
Age, mean (SD)	39.94 (13.04)	40.22 (12.90)	0.33	0.74	0.02
Gender, *n* (%)			0.04	0.85	0.006
Male	116 (28.5)	144 (29.1)			
Female	291 (71.5)	351 (70.9)			
Setting, *n* (%)			0.03	0.87	0.005
Hospital^a^	89 (21.9)	106 (21.4)			
On-line^b^	318 (78.1)	389 (78.6)			
Disease, *n* (%)			11.91	0.001	0.12
Ulcerative colitis	245 (60.2)	241 (48.7)			
Crohn's disease	162 (39.2)	254 (51.3)			
HADS, mean (SD)	20.56 (8.35)	17.71 (8.55)	5.02	<0.001	0.34
IBDQ, mean (SD)	119.99 (31.97)	152.91 (31.2)	15.56	<0.001	1.04
Bowel symptoms	38.56 (10.02)	50.69 (9.80)	18.30	<0.001	1.23
Emotional function	45.18 (12.41)	54.87 (12.61)	11.55	<0.001	0.77
Social function	19.93 (7.38)	26.22 (6.92)	13.15	<0.001	0.88
Systemic symptoms	16.31 (6.27)	21.24 (6.45)	11.56	<0.001	0.77
PHQ-12, mean (SD)	9.71 (4.49)	7.99 (4.29)	5.87	<0.001	0.39

a Patients recruited before Covid-19 pandemic;

b *Patients recruited during Covid-19 pandemic*.

*HADS, Hospital Anxiety and Depression Scale; IBDQ, Inflammatory Bowel Disease Questionnaire; PHQ-12, Patient Health Questionnaire-12*.

Predictors of HRQL were estimated through hierarchical regression models (Table 2). In the first model, the type of setting (i.e., patients recruited at the hospital care centers before *vs*. those recruited online during the Covid-19 lockdown) as a stand-alone variable explained only a small portion (8%), even though significant (β = −0.27, *p* < 0.001), of the IBDQ variance. The second model added age, gender and IBD-related clinical variables such as type of disease and disease activity. Disease activity (β = −0.48, *p* < 0.001) and, at a lesser extent, gender (β = −0.13, *p* < 0.001) and being affected by UC rather than CD (β = 0.13, *p* < 0.001) added a significant amount of explained IBDQ variance (25%). Independent prediction of poorer HRQL was further increased by 27% in the third model when psychological distress (β = −0.29, *p* < 0.001) and somatization (β = −0.36, *p* < 0.001) were added. The final model predicted 60% of the explained IBDQ variance, thus suggesting that half of the variance of IBD-related HRQL was due to the equal weight of active disease (particularly in UC patients) and psychological distress.

**Table 2:** Predicting HRQL (IBDQ total score as the dependent variable) from clinical characteristics and psychological symptoms.

	***R*^2^**	**ΔR^2^**	**B**	**SE**	**β**
Model 1	0.08	0.08			
Setting^*a*^			−23.96	2.81	−0.27^***^
Model 2	0.32	0.25			
Setting			−19.27	2.68	−0.22 ^***^
+ Age			−0.10	0.08	−0.04
+ Gender*^*b*^*			−10.40	2.35	−0.13^***^
+ Disease*^*c*^*			9.11	2.00	0.13^***^
+ Disease activity^d^			−34.00	1.98	−0.48^***^
Model 3	0.60	0.27			
Setting			−8.73	2.13	−0.10^***^
Age			−0.11	0.06	−0.04
Gender			1.41	1.90	0.02
Disease			6.25	1.56	0.09^***^
Disease activity			−25.28	1.58	−0.35^***^
+ HADS			−1.21	0.11	−0.29^***^
+ PHQ−12			−2.86	0.22	−0.36^***^

*** *p < 0.001*.

a *1 = Hospital, patients recruited before Covid-19 pandemic; 2 = Online, patients recruited during Covid-19 pandemic*.

b *1 = male; 2 = female*.

c *1 = Crohn's disease; 2= ulcerative colitis*.

d *1 = quiescent; 2 = active*.

*HADS, Hospital Anxiety and Depression Scale; IBDQ, Inflammatory Bowel Disease Questionnaire; PHQ-12, Patient Health Questionnaire-12*.

## Discussion

This preliminary cross-sectional study was conducted 2–3 months after the WHO-declaration about the pandemic. The main result is that while active IBD disease significantly affected psychological distress, somatization symptoms, and HRQL (as expected), the pandemic-related living condition played unexpectedly only a negligible role in the association with HRQL. In turn, poorer IBD-related HRQL was more strongly and independently predicted by clinical factors (particularly disease activity in UC patients) and psychological distress.

Only some few studies have previously reported the different effects of catastrophic events on the health outcomes of medical disorders showing controversial findings. For example, in a Japanese study, the incidence and in-hospital mortality rate of heart failure improved after the Great East Japan Earthquake (March 2011) (6). Another study showed a time-dependent effect of the severe psychological stress caused by that earthquake (7). On the short term, up to 1 month after the disaster, the rate of multiple peptic ulcers was about 3.5 times greater than in the previous year and, of note, none of those patients had positive H.pylori infection. Conversely, in the chronic stress phase (>1 month after the earthquake), the number of ulcer patients decreased to a level similar to that of the previous year. A similar time-dependent pattern was observed after the April 2009 earthquake in Abruzzo, Italy, in the hospitalization rates for respiratory diseases (pneumonia, chronic obstructive pulmonary disease, and respiratory insufficiency) (9). In the short-term within the first 6 months, there was a significant increase in hospitalizations for pneumonia and respiratory insufficiency in the geographical earthquake area compared to neighbor areas. Conversely, in medium-long term (up to 5 years), a significant difference on the slope decrease of hospitalizations for acute and chronic respiratory diseases in the earthquake vs. the neighbor geographical areas. Consistently, also studies investigating the incidence rates of IBD flare-ups after the Great East Japan Earthquake showed an increase in the short follow-up period (after 4 months) and a normalization within the expected rates in the long run (after 24 months) (21, 22).

Although there have been several studies on the psychological effects of the Covid-19 pandemic (35), no data on the quality of life of IBD patients during the stay-at-home period is available. Some few investigations showed that HRQL decreased significantly in patients with cardiovascular diseases (36) and cancer waiting for surgical intervention that was postponed and not rescheduled because of the Covid-19 epidemic (37). Messages in the media that strongly supported social distancing during the lockdown period may have likely hindered access to health care services of chronic medical patients, including IBD. Furthermore, widespread health-related concerns increased during the first wave of Covid-19 pandemic, either related directly to the coronavirus-specific symptom perception (38) and indirectly to higher somatic amplification (39). In addition, IBD patients treated with systemic corticosteroids and sulfasalazine or five-aminosalicylate were at significant higher risk for severe Covid-19 symptoms (40), thus inducing higher health anxiety in these patients. Finally, health anxiety might have been further aggravated by early beliefs of higher risk for coronavirus infection in chronic patients treated with immunosuppressants, even though immunomodulators have been shown to have neutral or even protective antiviral effects in IBD patients (40, 41).

In our study, while active IBD disease significantly affected psychological distress, somatization symptoms, and HRQL (as expected), the pandemic-related living condition played unexpectedly only a negligible role in the association with HRQL. Consistent with one of the few previous investigations (23), self-isolation during the Covid-19 outbreak seems to be less linked with impaired HRQL than the clinical condition (particularly disease activity) and psychological distress of the patients. This is an interesting result, given that being in isolation could decrease the discomfort causing by the management of the symptomatology and the fear of contracting Covid-19 in these patients.

Our findings are consistent with previous literature showing significant empirical and clinical relationships between psychological distress, HRQL, and disease activity within the framework of the brain-gut axis model (14, 42). There is growing evidence that psychological distress can predict disease gravity and severe relapses and that this relationship is bidirectional. A systematic review has shown that depressive symptoms could get up to 40% during active disease (43). This has also been found in another study which showed the psychological distress was dependent upon the disease activity, being improved in those with active disease compared to those in a state of remission (44). In a further study, it was reported that the presence of anxiety and depression was strictly related to disease relapses (45). Furthermore, among patients with IBD, a higher level of perceived stress is a strong predictor of lower HRQL and lower adherence to provider recommendations (46). On the other hand, HRQL has been recognized as a key factor in the care of patients with IBD (14).

Some limitations are to be acknowledged. Firstly, the cross-sectional nature of our data does not allow us to establish the direction of causality as well as the timing and sequence of symptom onset during the pandemic. The current survey was conducted soon after the WHO declaration of Covid-19 pandemic and during the acute epidemic peak of the first wave. Further longitudinal studies are needed to investigate the long-term effects of this new catastrophic event on psychological health, HRQL, and the disease course of IBD patients. To this purpose, a follow-up online survey has been planned by our research group. Secondly, the online administration of a survey is subject to responder bias, because patients with lower education or older age are less likely to participate in the Internet research. Third, many possible mediating variables were not assessed and could not be controlled for, as inflammation status, diet, and personality traits; furthermore, an appropriate psychological assessment related to the Covid-19 associated living conditions' stress is necessary to evaluate whether the stress of covid-19 lockdown actually did not contribute to the psychological state and clinical disease manifestation during this period. Lastly, self-reported disease activity, could not be checked against the actual medical data.

In conclusion, our daily lives have been transformed by the Covid-19 pandemic and its related lockdowns, leading to alarming psychological consequences. Since healthcare institutions will have to deal with Covid-19 and its psychological consequences for many months to come, a thorough understanding of the medium and long-term effects of pandemic on patients with chronic disease is needed to mitigate the traumatic impact and to develop appropriate support interventions.

In our study, clinical and psychological manifestations seem to be major impairments in IBD patients both before and during the Covid-19 outbreak. Furthermore, IBD patients seem to be more concerned by bowel, emotional, social, and systemic symptoms (IBDQ subscales) and psychological and somatizing distressing symptoms than the pandemic-related living conditions. This lends further weight to the need of developing an evidence-based, integrated, biopsychosocial model of care for patients with IBD to disentangle subjective and objective factors that affect the burden of disease.

## Data Availability Statement

The raw data supporting the conclusions of this article will be made available by the authors, without undue reservation.

## Ethics Statement

The studies involving human participants were reviewed and approved by INSTITUTIONAL REVIEW BOARD OF PSYCHOLOGY (IRBP) DEL DIPARTIMENTO DI SCIENZE PSICOLOGICHE, DELLA SALUTE E DEL TERRITORIO (DiSPuTer)—UNIVERSITÀ DEGLI STUDI DI CHIETI-PESCARA. The patients/participants provided their written informed consent to participate in this study.

## Author Contributions

CC and IR wrote the original draft, conducted statistical analysis, and provided substantial contributions to the conception and design of the paper. LZ wrote the original draft by revising it critically for important intellectual content. LG, MN, and KE are the physicians contributed to collected data. PP conceived the research and wrote the final version of the paper. Finally, all the authors have approved the final version of the manuscript and were accountable for the content of the work.

## Conflict of Interest

The authors declare that the research was conducted in the absence of any commercial or financial relationships that could be construed as a potential conflict of interest.
